# Development of gene-based molecular markers tagging low alkaloid *pauper* locus in white lupin (*Lupinus albus* L.)

**DOI:** 10.1007/s13353-019-00508-9

**Published:** 2019-08-13

**Authors:** Sandra Rychel, Michał Książkiewicz

**Affiliations:** Institute of Plant Genetics, Polish Academy of Sciences, Strzeszyńska 34, 60-479 Poznań, Poland

**Keywords:** White lupin, Alkaloids, Molecular markers, Linkage mapping

## Abstract

White lupin (*Lupinus albus* L.) is a legume grain crop cultivated since ancient Greece and Egypt. Modern white lupin cultivars are appreciated as a source of protein with positive nutraceutical impact. However, white lupins produce anti-nutritional compounds, quinolizidine alkaloids, which provide bitter taste and have a negative influence on human health. During domestication of this species, several recessive alleles at unlinked loci controlling low alkaloid content were selected. One of these loci, *pauper*, was exploited worldwide providing numerous low-alkaloid cultivars. However, molecular tracking of *pauper* has been hampered due to the lack of diagnostic markers. In the present study, the synteny-based approach was harnessed to target *pauper* locus. Single-nucleotide polymorphisms flanking *pauper* locus on white lupin linkage map as well as candidate gene sequences elucidated from the narrow-leafed lupin (*L. angustifolius* L.) chromosome segment syntenic to the *pauper* linkage group region were transformed to PCR-based molecular markers. These markers were analyzed both in the mapping population and world germplasm collection. From fourteen markers screened, eleven were localized at a distance below 1.5 cM from this locus, including five co-segregating with *pauper*. The linkage of these markers was confirmed by high LOD values (up to 58.4). Validation performed in the set of 127 bitter and 23 sweet accessions evidenced high applicability of one marker, LAGI01_35805_F1_R1, for *pauper* locus selection, highlighted by the low ratio of false-positive scores (2.5%). LAGI01_35805 represents a homolog of *L. angustifolius* acyltransferase-like (*LaAT*) gene which might hypothetically participate in the alkaloid biosynthesis process in lupins.

**Electronic supplementary material:**

The online version of this article (10.1007/s13353-019-00508-9) contains supplementary material, which is available to authorized users.

## Introduction

White lupin (*Lupinus albus* L.) is a cool season grain legume crop with a relatively long history of cultivation. Primary domestication of *L. albus* has occurred in ancient Greece and Egypt to produce grain for human and animal consumption as well as in ancient Rome as green manure (Gladstones 1970). Lupins were found to be very beneficial in crop rotations because they increase soil fertility through symbiotic nitrogen fixation and efficient mobilization of soil phosphorus (Lambers et al. 2013). Modern lupin cultivars are appreciated also as a valuable source of protein (38–42% in seeds) (Papineau and Huyghe 2004) with positive nutraceutical impact on hypercholesterolemia, hypertension, and hyperglycemia (Arnoldi and Greco 2011). Moreover, white lupin crops have moderate seed content of oil (10–13%) with desirable ratios of omega-6 to omega-3 acids for consumption purposes (Boschin et al. 2007). These advantages make this species valuable for human food and animal feed (Lin et al. 2009). However, white lupin seeds contain some content of anti-nutritional compounds, including quinolizidine alkaloids (up to 12% in wild populations) and oligosaccharides (up to 10%) (Kroc et al. 2017; Mohamed and Rayas-Duarte 1995). Average total alkaloid content in white lupin breeding lines and cultivars is about 1.3% of the seed dry weight; however, in some sweet accessions, these values are below 0.02% (Kroc et al. 2017).

Alkaloids are considered as the major unfavorable components in white lupin due to their bitter taste and negative influence on human health, causing in the worst-case scenario acute anticholinergic toxicity (Daverio et al. 2014). Therefore, during the domestication process, numerous efforts aiming at the reduction of alkaloid levels were strongly emphasized. As many as nine hypothetical loci controlling low alkaloid content were initially identified throughout white lupin breeding, including *pauper*, *primus*, *tercius*, *exiguus*, *nutricius*, *mitis*, *suavis*, *reductus*, and *minutus* (Hackbarth 1957; Hackbarth 1961; Porsche 1964; Šatović 1993; Troll 1958). However, *primus* and *tercius* were identified as the synonyms of *pauper*, whereas *suavis* and *minutus* were not studied as extensively as the other loci and their independence cannot be authenticated (Harrison and Williams 1982). Three low-alkaloid alleles originating from different loci were introduced into white lupin cultivars in the early years of modern domestication, namely *exiguus* (cv. Neuland, 1937), *pauper* (cv. Kraftquell, cv. Ultra, and cv. Przebędowski Wczesny, 1949–1950), and *nutricius* (cv. Nahrquell, 1949); nevertheless, only a *pauper* was exploited worldwide (Harrison and Williams 1982; Šatović 1993).

To facilitate molecular studies on white lupin domestication genes, a recombinant inbred line (RIL) mapping population was developed, descending from parental cross between Kiev Mutant (Ukrainian cv, sweet, early flowering, anthracnose susceptible) and P27174 (Ethiopian landrace, bitter, late flowering, anthracnose resistant) (Phan et al. 2007). A linkage map carrying 220 amplified fragment length polymorphism (AFLP) and 105 PCR-amplified gene–based markers was developed for this RIL population, but the distance between the *pauper* locus and flanking markers was revealed to be higher than 20 cM in both directions (Phan et al. 2007). This map was later updated with the set of 136 diversity array technology (DArT) markers but no improvement around the *pauper* locus was achieved as the flanking markers were split into two separate linkage groups (Vipin et al. 2013). With the aid of the microsatellite-anchored fragment length polymorphism (MFLP) technique, the PauperM1 marker was developed, which was confirmed in the Kiev Mutant × P27174 RIL population to be linked to the *pauper* locus at the genetic distance of 1.4 cM (Lin et al. 2009). However, this marker required sequencing gel and radioisotope primer labelling for the correct determination of alleles. Moreover, the applicability of this marker was restricted to ~ 95% of bitter lines and 91% of sweet non-pauper lines (Lin et al. 2009). Recently, a high-density consensus linkage map of white lupin genome was constructed, which integrated 453 published markers with 3597 newly developed sequence-based markers and constituted a single linkage group per every chromosome (Książkiewicz et al. 2017). This map yielded several new markers co-segregating or localized closer to the *pauper* locus than the PauperM1. Moreover, this new reference linkage map anchored recently published transcriptome assembly (O’Rourke et al. 2013) to particular markers, and aligned these markers to syntenic blocks of the narrow-leafed lupin (*L. angustifolius* L.) genome sequence (Hane et al. 2017), providing novel opportunities for tracking white lupin domestication genes by comparative mapping approach.

In the present study, these resources were harnessed to analyze the genome region carrying low-alkaloid *pauper* locus. PCR-based markers were developed and implemented for the screening of white lupin germplasm collection carrying a diversified subset of sweet and bitter lines. The applicability of newly developed markers for *pauper* allele selection has been evaluated.

## Materials and methods

### Plant material

Genetic mapping was performed using the reference *L. albus* Kiev Mutant × P27174 recombinant inbred line (RIL) population (F_8_, *n* = 195), delivered by the Department of Agriculture and Food, Western Australia. This population was derived from a cross between a bitter, late flowering, and anthracnose-resistant Ethiopian landrace (P27174) and a sweet, early flowering, and anthracnose susceptible Ukrainian cultivar (Kiev Mutant) (Książkiewicz et al. 2017; Phan et al. 2007; Vipin et al. 2013).

The set of 160 *L. albus* lines derived from the European Lupin Gene Resources Database maintained by Poznań Plant Breeders Ltd. station located in Wiatrowo was used for marker validation: 79 primitive populations, 36 landraces, 30 cultivars, 12 cross derivatives, and 3 mutants. These lines originated from 23 countries. Taking into consideration alkaloid content in seeds, 127 lines were bitter (above 0.5% of total dry weight alkaloid content), 26 were sweet (below 0.2%), and 7 intermediate (Kroc et al. 2017) (Supplementary File 1).

Plants were grown in a greenhouse at the Institute of Plant Genetics of the Polish Academy of Sciences in Poznań under ambient long-day photoperiod (14–16 h). Leaves were collected from 4-week plants. DNA was isolated using DNeasy Plant Mini Kit (Qiagen, Hilden, Germany).

### Development of PCR-based markers

Marker sequences surrounding pauper locus (Książkiewicz et al. 2017) were aligned to the transcriptome datasets of Kiev and P27174 lines (Książkiewicz et al. 2017) as well as to the reference white lupin gene index LAGI01 (O’Rourke et al. 2013) by BLAST (Altschul et al. 1990) using Geneious software (Kearse et al. 2012). Matching transcripts were then comparatively mapped to the genome sequence of the narrow-leafed lupin (Hane et al. 2017), extracting selected loci with 10000 nt of flanking regions. To find exon/intron boundaries, white lupin marker and transcript sequences were assembled together with extracted narrow-leafed lupin genome regions into contigs using a progressive Mauve algorithm (Darling et al. 2004) assuming genome collinearity. Mauve alignments carrying markers, corresponding white lupin transcripts and fragments of narrow-leafed lupin scaffolds, were searched for the presence of polymorphic loci. Primers flanking these loci were designed using Primer3Plus (Untergasser et al. 2007).

PCR was performed using Labcycler thermocycler (SensoQuest GmbH, Göttingen, Germany). GoTaq® G2 Flexi DNA Polymerase (Promega) was used. The reaction mixture contained DNA template (2.5 ng/μL), dNTP (0.8 mM), MgCl_2_ (2 mM), polymerase (1 U), polymerase buffer (1×), and primers (0.25 μM each).

PCR amplicons were purified directly from the post-reaction mixtures (QIAquick PCR Purification Kit; Qiagen) and sequenced (ABI PRISM 3130 XL Genetic Analyzer; Applied Biosystems, Hitachi) in the Laboratory of Molecular Biology Techniques, Faculty of Biology, Adam Mickiewicz University (Poznan, Poland). Cleaved amplified polymorphic sequence (CAPS) (Konieczny and Ausubel 1993) or derived CAPS (dCAPS) (Neff et al. 1998) approaches were used to resolve the nucleotide substitution polymorphisms. Restriction sites and dCAPS primers were identified using dCAPS Finder 2.0 (Neff et al. 2002) and SNP2CAPS (Thiel et al. 2004). Restriction products were separated by agarose gel electrophoresis, with the agarose concentration (1–3%) adjusted to follow the size of the expected digestion products.

### Linkage mapping

Chi-square (*χ*^2^) values for Mendelian segregation in F_8_ RILs were estimated using the expected 1:1 segregation ratio. The calculation of probability was based on *χ*^2^ and 2 degrees of freedom. *L. albus* marker segregation files (Książkiewicz et al. 2017; Phan et al. 2007; Rychel et al. 2019; Vipin et al. 2013), together with those developed in this study, were imported to Joinmap 5.0 (Stam 1993). Mapping procedure was performed as previously described (Książkiewicz et al. 2017). Based on the initial results of the mapping, the line RIL-169 was removed from the final mapping due to the frequent change of marker allelic phases. Such an observation may result from seed admixture or cross-pollination during seed multiplication. Twenty repeats of linkage group calculation with altered parameters were done to estimate the plausibility of marker positions. LOD values were calculated in Map Manager QTXb20 (Manly et al. 2001).

### Marker validation

Markers were validated by comparing seed dry weight alkaloid content (Kroc et al. 2017) and marker allelic phases for the set of 160 collection lines. The Pearson product-moment correlation coefficient was calculated in Excel. Taking into consideration the hypothesis on the influence of a single gene (in the *pauper* locus) on the alkaloid content, binary data similarity analysis was performed. Therefore, alkaloid content values above 0.2% were assigned as 1 (bitter and intermediate) and the remaining values as 0 (sweet), however, known as non-*pauper* low alkaloid lines were assigned as 1 to avoid unjustified false-negative results. Kiev Mutant-like scores were assigned as 0, P27174-like scores as 1, and heterozygotes also as 1 because *pauper* is a recessive allele. Simple matching (Sokal and Michener 1958) and Rogers-Tanimoto (Rogers and Tanimoto 1960) coefficients were calculated using binary similarity calculator http://www.minerazzi.com/tools/similarity/binary-similarity-calculator.php.

## Results

### Selection of sequences from *pauper* locus

Molecular markers from the most recent *L. albus* linkage map localized in the proximity of the *pauper* locus were aligned to the *L. angustifolius* genome and gene sequences (Hane et al. 2017) as well as to the *L. albus* transcriptome (O’Rourke et al. 2013) to find anchors for primer design. Based on the alignments and positions on the linkage map, 6 markers were selected (TP16854, TP22150, TP30216, TP309728, TP447859, TP70046) for further study. One of these markers, TP16854, matched Lup021586 gene, which was shown to have 100% nucleotide identity to the *LaAT* gene (AB581532.1) encoding acyltransferase-like protein (Książkiewicz et al. 2017). As the *LaAT* gene expression was revealed to be correlated with total alkaloid content (Bunsupa et al. 2011), it was included in the marker array. When the *LaAT* was blasted against *L. albus* transcriptome, it tagged LAGI01_35805 and LAGI01_49436 as the most similar sequences. These transcripts were found to have high similarity to *L. angustifolius* Lup021586 and Lup021583 genes, annotated as encoding HXXXD-type acyltransferase family proteins. Analysis of the surrounding genes in the *L. angustifolius* assembly highlighted a Lup021589 (a bHLH35-like transcription factor) as a another hypothetical candidate for marker development. Mapping of the Lup021589 to the *L. albus* transcriptome resulted in the selection of LAGI01_54458. To summarize, the set of sequences used for *pauper* marker development consisted of TP16854, TP22150, TP30216, TP309728, TP447859, TP70046, LAGI01_35805, LAGI01_49436, and LAGI01_54458.

### Development of PCR-based *pauper* molecular marker array

For TP16854, TP22150, TP30216, TP309728, TP447859, and TP70046 sequences, markers were developed using those polymorphic loci which were published in the linkage map paper (Książkiewicz et al. 2017). Two dCAPS (TP16854_FD_R and TP70046_F_RD) and four CAPS (TP22150_F_R, TP30216_F_R, TP309728_F_R, TP447859_F_R) markers were designed. Mapping Kiev Mutant and P27174 reads (PRJNA380248) to the *L. albus* transcriptome revealed polymorphic loci in one exon in LAGI01_49436 and LAGI01_54458, and in two exons in LAGI01_35805. Three CAPS markers (LAGI01_35805_F1_R1, LAGI01_35805_F2_R2, LAGI01_49436_F2_R2) and one PCR-based INDEL marker (LAGI01_54458_F2_R1) were designed. Initial screening of white lupin lines with published PauperM1 marker (Lin et al. 2009) revealed high difficulty of inferring allele genotypes due to very small difference in product length and amplification of stutter bands. Therefore, this marker was transformed into a pair of CAPS markers exploiting two different polymorphic loci. The marker array was supplemented by two gene-based markers, ESD4-F7 and ESD4-F8, which were recently developed for *L. albus* homolog of *A. thaliana* flowering induction pathway gene EARLY IN SHORT DAYS 4 (ESD4) and mapped very close to the *pauper* locus (Rychel et al. 2019). The list of developed markers with information on assigned *L. angustifolius* gene and genome sequences and *L. albus* transcripts is provided in Table 1. The list of primer pairs, PCR primer annealing temperature, enzyme used for polymorphism detection, and the lengths of restriction products for Kiev Mutant and P27174 lines are provided in Table 2.

**Table 1:** Sequences assigned to developed white lupin markers tagging *pauper* locus. Positions indicate the first nucleotide in the marker sequence alignment

Marker	*L. angustifolius* chromosome	Position (nt)	*L. angustifolius* gene	Position (nt)	*L. albus* transcript	Position (nt)
ESD4-F7^a^	NLL-16	19 043 934	Lup021600	1	LAGI01_22606	1580
ESD4-F8^a^	NLL-16	19 043 385	Lup021600	417	LAGI01_23187	1003
LAGI01_35805_F1_R1	NLL-16	18 720 613	Lup021586	2	LAGI01_35805	149
LAGI01_35805_F2_R2	NLL-16	18 718 376	Lup021586	231	LAGI01_35805	378
LAGI01_49436_F2_R2	NLL-16	18 680 802	Lup021583	804	LAGI01_49436	71
LAGI01_54458_F2_R1	NLL-16	18 798 603	–	–	LAGI01_54458	538
PauperM1_F_R^b^	NLL-16	10 380 713	–	–	–	–
TP16854_FD_R	NLL-16	18 718 404	Lup021586	730	LAGI01_35805	880
TP22150_F_R	NLL-16	19 032 414	Lup021599	1050	LAGI01_34026	56
TP30216_F_R	NLL-16	9 710 799	Lup030539	591	LAGI01_22438	1175
TP309728_F_R	NLL-16	19 185 829	Lup021606	773	LAGI01_61485	673
TP447859_F_R	NLL-16	19 092 892	Lup021602	389	LAGI01_9900	557
TP70046_F_RD	NLL-19	15 265 260	Lup026514	403	LAGI01_45076	417

^a^Marker published by (Rychel et al. 2019)

^b^Marker published by (Lin et al. 2009)

**Table 2:** List of developed markers tagging *pauper* locus, with primer sequences, PCR primer annealing temperature, validated enzymes, and restriction product sizes

Name	Primers	PCR (°C)	Validated enzyme	Products Kiev (bp)	Products P27174 (bp)
TP16854_FD_R	CAGCAAGAGATTCAAATGTTGTGAAAG ATGAAGGAAATTAGTTGCAATGAATCAA	60	*Hin*dIII	64	39, 25
TP447859_F_R	CTGCTTCTGATATTTTCAATGCATT AAGCCATCAAATGCTTCAGAAGTAG	60	*Mnl*I	56, 17	73
TP22150_F_R	TTGCATGAGGGAAGATGGACG ATAGCATGTGACCTGGAGGAC	60	*Mnl*I	81, 48, 25, 8	129, 25, 8
TP309728_F_R	TTGAAGGTCTACAAAGGCATCC TATAGACCAGCACAACCCTCTG	60	*Taq*I	75, 40	115
TP70046_F_RD	CCATCATTCCTGCCATAAGG ATCAAGGAAGAGTTAGAGAAT	60	*Hin*fI	110, 21	131
TP30216_F_R	TATTAAGTCGATTTGGTGGAGCT AATACATGAAATAGGCCCAATGAAA	56	*Hin*fI	76, 34	110
LAGI01_49436_F2_R2	CTCTGAACTTGGGCATGGATG ACTTGGTGCCTATGTATGGAGA	64	*Bam*HI	464, 66	527
LAGI01_35805_F1_R1	TGGCATCACTGAAAATTGAGATGA CTTTGAGAGGGCTTGTTTGAG	64	*Bcl*I	197	108, 89
LAGI01_35805_F2_R2	GCTAGTAAAACAACCCAACGGA GCCCTAGCTCTTGATCTCCAG	64	*Mbo*I	253, 216, 109, 16	362, 216, 16
LAGI01_54458_F2_R1	AAAAGTGTTAGAAAAGTACAGCAC CAGTGACTCCAACAAAAGCACA	52	–	108	98
ESD4-F8^a^	CTGCGCTTCTCCTGTAGTTCAATTT AGACCTTGAAACCGATAGTCATAATAG	63	*Mse*I	351	178, 173
ESD4-F7^a^	CCCTAACCAACGGTAGCTTGATAT TGTGCTTTTTCACCCCTCTC	52	*Eco*RV	232, 22	254
PauperM1_F_R_HhaI^b^	AAGAAAAGGCCCAATG TTTTAAAGTCATACCATTGAG	54	*Hha*I	~ 209	108, 99
PauperM1_F_R_HinfI^b^	AAGAAAAGGCCCAATG TTTTAAAGTCATACCATTGAG	54	*Hin*fI	145, ~ 64	207

^a^Marker published by (Rychel et al. 2019)

^b^Improvement of the PauperM1 marker published by (Lin et al. 2009)

### Linkage mapping of *pauper* markers

**Fig. 1:**
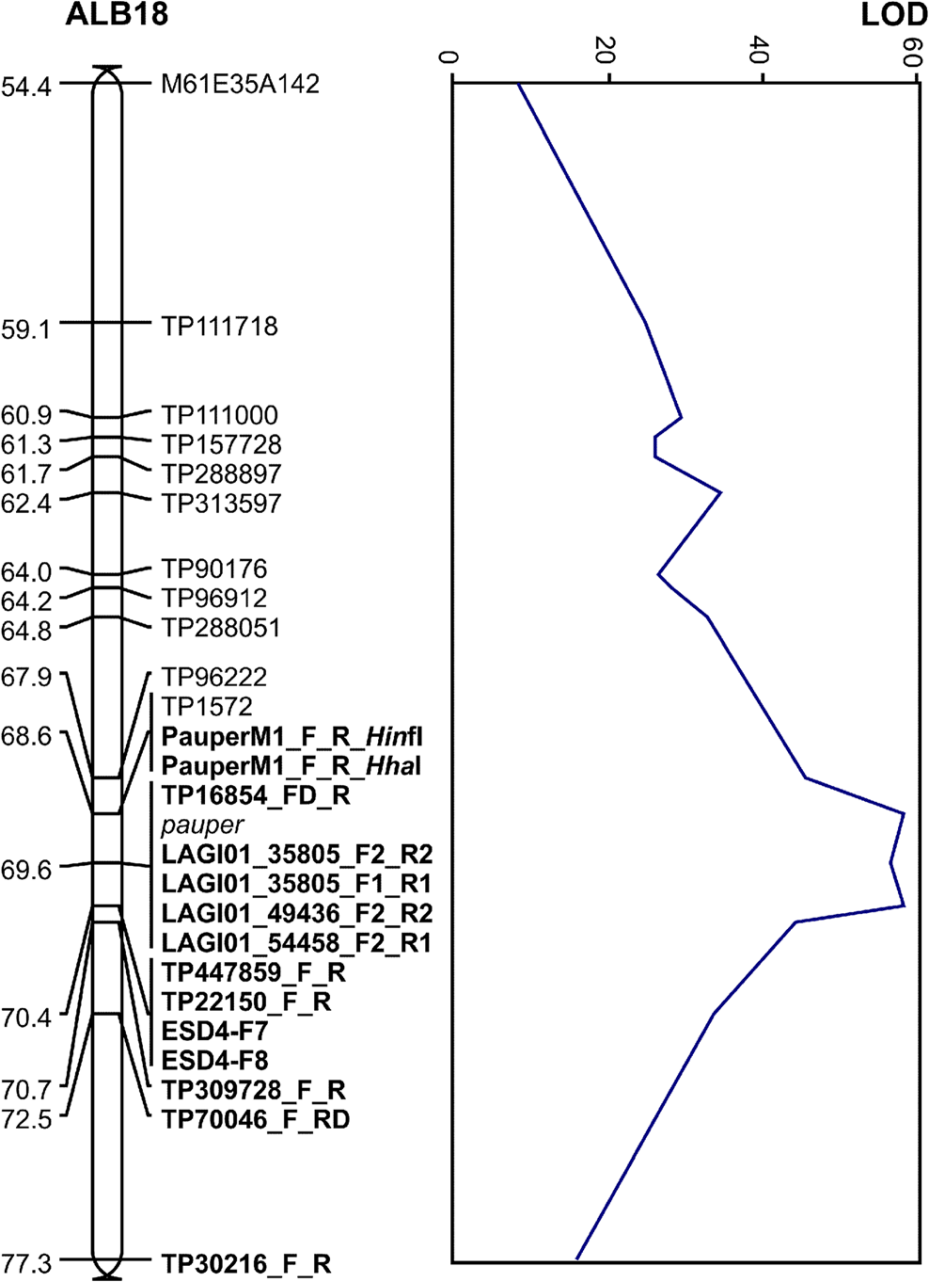
Localization of markers in the white lupin linkage group ALB18 region carrying *pauper* locus. Vertical bar graph visualizes linkage group fragment with cumulative genetic distance scale expressed in centimorgans (cM). The linear plot shows corresponding LOD values of linkage to adjacent markers. Names of markers analyzed in this study are in boldface

The segregation of newly developed gene-based markers (LAGI01_49436_F2_R2, LAGI01_35805_F1_R1, LAGI01_35805_F2_R2, LAGI01_54458_F2_R1) was analyzed in the RIL population to provide data for linkage mapping. Markers PauperM1, TP16854, TP447859, TP22150, TP309728, TP70046, and TP30216 were already localized on the genetic map (Książkiewicz et al. 2017), however, with missing 14–53% of RIL genotyping data. To increase the quality of linkage mapping, the segregation of these markers was tested as well. Segregation data were obtained for 98.5% of RILs. All markers were localized in the linkage group ALB18 in the region carrying *pauper* locus. Both PauperM1 markers revealed identical segregation and localized 1.06 cM upstream the *pauper* locus. Five markers (TP16854_FD_R, LAGI01_35805_F2_R2, LAGI01_35805_F1_R1, LAGI01_49436_F2_R2, and LAGI01_54458_F2_R1) co-segregated with the *pauper* locus (Fig. 1). The linkage of these markers was confirmed by high-LOD values (min 49.1, max 56.6, mean 55.25). Markers TP447859_F_R, TP22150_F_R, ESD4-F7, and ESD4-F8 formed a redundant cluster localized 0.79 cM downstream the *pauper* locus. Markers TP309728_F_R, TP70046_F_RD, and TP30216_F_R were mapped at further distances from the *pauper*, namely 1.06 cM, 2.91 cM, and 7.70 cM. Information on marker *χ*^2^*P* values for segregation distortion, position in linkage group, and LOD values is provided in Table 3. RIL segregation data is given in Supplementary File 2.

**Table 3:** RIL segregation data, chi-square *P* values for segregation distortion, linkage group positions, and LOD scores to adjacent loci calculated for white lupin *pauper* markers

Name	% RIL data	*χ* ^2^ *P* value	Linkage group	Locus (cM)	LOD scores
PauperM1_F_R_HhaI	99.0	0.75	ALB18	68.57	52.7, 58.1
PauperM1_F_R_HinfI	99.0	0.75	ALB18	68.57	58.1, 49.1
*pauper* ^a^	95.4	0.66	ALB18	69.64	55.4, 54.8
LAGI01_49436_F2_R2	96.4	0.76	ALB18	69.64	56.3, 56.3
TP16854_FD_R	99.0	0.83	ALB18	69.64	49.1, 55.4
LAGI01_35805_F2_R2	97.4	0.78	ALB18	69.64	54.8, 56.6
LAGI01_35805_F1_R1	98.0	0.71	ALB18	69.64	56.6, 56.3
LAGI01_54458_F2_R1	98.0	0.83	ALB18	69.64	56.3, 54.8
TP22150_F_R	99.5	0.78	ALB18	70.43	58.1, 58.4
ESD4-F7	99.5	0.78	ALB18	70.43	58.4, 58.4
ESD4-F8	99.5	0.78	ALB18	70.43	58.4, 55.1
TP447859_F_R	99.0	0.83	ALB18	70.43	54.8, 58.1
TP309728_F_R	98.5	0.78	ALB18	70.69	55.1, 44.2
TP70046_F_RD	96.4	0.83	ALB18	72.55	44.2, 33.6
TP30216_F_R	99.5	0.25	ALB18	77.34	33.6, 16.0

^a^Locus published by (Książkiewicz et al. 2017; Phan et al. 2007)

### *Pauper* marker validation in collection lines

Validation set of lines contained 127 bitter and five *pauper* (Kiev Mutant, Boros, Lotos, Hansa, Kali), four *exiguus* (Start, Nelly USA, Butan, Tombowskij Skorospielyj), one *nutricius* (Nahrquell), and sixteen other sweet or intermediate accessions with unknown genotype according to the information obtained from the Plant Breeding Smolice Ltd., Co. (Dr. Stanisław Stawiński) and published data (Hackbarth 1957; Hackbarth 1961; Harrison and Williams 1982; Kroc et al. 2017; Porsche 1964; Šatović 1993; Troll 1958). Visualization of marker polymorphism for selected lines is provided in Supplementary File 3. Simple matching coefficients were calculated, to compare the marker genotype and the *pauper* phenotype. These values ranged from 0.15 to 0.94, indicating rapid linkage disequilibrium decay around *pauper* locus. To address the putative applicability of newly developed markers in the marker-assisted selection, Rogers-Tanimoto coefficient values were calculated. Rogers-Tanimoto is a variant of the simple matching coefficient that gives double weight to mismatching variables, therefore accentuating false-positive and false-negative scores. Rogers-Tanimoto values were in the range from 0.08 to 0.88. LAGI54458_F1 marker revealed to have identical simple matching and Rogers-Tanimoto values as the previously published PauperM1, namely 0.83 and 0.71. LAGI01_35805_F1_R1 marker having those values as high as 0.94 and 0.88 was evidenced to be more applicable to marker-assisted selection than the PauperM1 (Table 4). All lines carrying *pauper* recessive alleles revealed positive LAGI01_35805_F1_R1 marker score. False-positive scores were obtained for four lines, namely 95015 “San Felices” (12.73% alkaloid dry weight content), 95064 “Population-8062” (2.69%), 95220 “FAM 120” (1.21%), and 95023 “Oeiras-930/3” (4.93%) (Kroc et al. 2017). All these lines are primitive accessions or landraces. Marker scores for the set of validation lines are provided in Supplementary File 4.

**Table 4:** Validation of *pauper* markers in white lupin collection lines differing in alkaloid content

Marker	Simple matching coefficient with bitter/sweet score	Rogers-Tanimoto coefficient with bitter/sweet score	Correlation with bitter/sweet score	Correlation with % alkaloid content
LAGI01_35805_F1_R1	0.94	0.88	0.71	0.26
LAGI01_54458_F2_R1	0.83	0.71	0.41	0.15
PauperM1_F_R_HhaI	0.83	0.71	0.45	0.15
PauperM1_F_R_HinfI	0.83	0.71	0.45	0.15
TP70046_F_RD	0.68	0.52	0.33	0.27
TP16854_FD_R	0.64	0.47	0.34	0.31
TP30216_F_R	0.52	0.35	0.18	0.09
TP309728_F_R	0.49	0.33	0.09	0.02
TP447859_F_R	0.47	0.30	0.07	0.06
LAGI01_35805_F2_R2	0.45	0.29	0.25	0.26
LAGI01_49436_F2_R2	0.31	0.18	0.17	0.16
TP22150_F_R	0.18	0.10	0.03	− 0.05
ESD4-F8	0.15	0.08	0.05	− 0.01
ESD4-F7	0.15	0.08	0.05	− 0.01

## Discussion

### Exploitation of shared synteny for genetic studies in legumes

In the present study, the synteny-based approach was applied to design molecular markers delimiting the *pauper* locus. The concept of exploitation of genome collinearity to transfer information from model plants to crop species emerged soon after sequencing the first three legume species (Cannon et al. 2009; Mudge et al. 2005). One of the first examples was positional cloning of a legume symbiosis *LjSym2* gene based on the comparative mapping between the three genomes differing by the advancement of molecular tools developed, namely *Lotus japonicus* (linkage map and contigs of transformation-competent artificial chromosomes), *Pisum sativum* (linkage map), and *A. thaliana* (chromosome-scale genome assembly) (Stracke et al. 2004). The sequence of *A. thaliana* genome revealed to be beneficial in mapping candidate genes conferring important agronomic domestication traits, growth determination, and photoperiod sensitivity, in common bean (Kwak et al. 2008). Molecular and comparative mapping combined with classical genetic approach was successfully applied to decipher the gene underlying yellow/green cotyledon polymorphism, which was first reported by Gregor Mendel in 1866 (Armstead et al. 2007). In the early years of legume comparative genomic studies, map-based cloning strategy resulted in the identification of the *RCT1*, an *M. truncatula* resistance gene that confers the multi-race resistance of alfalfa to a hemibiotrophic fungal pathogen *Colletotrichum trifolii*, causing anthracnose disease (Yang et al. 2008b). Progress in legume genome sequencing provided novel evidence for large-scale synteny existing between the papilionoid subclades which diverged about 50 million years ago (Bertioli et al. 2009). When the genome assemblies of *M. truncatula* and *L. japonicus* were aligned to the linkage map of *Phaseolus vulgaris*, novel large-scale macrosyntenic blocks were identified, justifying the concept of cross-species comparisons for tracking particular domestication genes (McConnell et al. 2010). As an example, a candidate gene for the hypernodulation mutation *nod3* in pea was elucidated by the comparative mapping to *M. truncatula* genome carrying a nodulation regulation *Pub1* gene in the syntenic region (Bordat et al. 2011). To facilitate studies involving genome sequence comparisons and synteny-based gene annotation, a LegumeIP 2.0 platform hosting large-scale genomic/transcriptomic data and integrative tools for bioinformatic analysis has been launched (Li et al. 2012a; Li et al. 2016). The reconstruction of a comparative map composed of seven species from the galegoid clade (*M. truncatula*, *M. sativa*, *Lens culinaris*, *P. sativum*, *L. japonicus*, *Cicer arietinum*, *Vicia faba*) and three species from the phaseoloid clade (*Vigna radiata*, *P. vulgaris*, *Glycine max*) carrying cross-species gene-derived markers revealed numerous macrosyntenic segments shared between all species analyzed (Lee et al. 2017). Recently, ten sequenced legume genomes were hierarchically aligned to establish a family-level genomics platform for studying evolutionary changes as well as functional analysis of genes involved in regulatory pathways (Wang et al. 2017).

Among lupins, *L. angustifolius* was the first species subjected to genetic map development and physical mapping studies (Boersma et al. 2005; Kaczmarek et al. 2009; Kasprzak et al. 2006; Kruszka and Wolko 1999; Leśniewska et al. 2011; Nelson et al. 2006). Comparative mapping of the progressively improved versions of *L. angustifolius* linkage map to sequence legume genomes revealed multiple blocks of conserved synteny carrying gene-rich regions and some candidate domestication genes (Kamphuis et al. 2015; Kroc et al. 2014; Książkiewicz et al. 2016; Książkiewicz et al. 2013; Książkiewicz et al. 2015; Nelson et al. 2010; Nelson et al. 2006; Przysiecka et al. 2015; Wyrwa et al. 2016). The synteny-based approach was further exploited to identify the particular gene (*LanFTc1*, a homolog of *A. thaliana FLOWERING LOCUS T*) underlying one of the major domestication loci in *L. angustifolius*, *Ku*, conferring vernalization independence and early flowering (Nelson et al. 2017; Nelson et al. 2006). Even the mechanism of regulation, based on the relatively long insertion/deletions in the promoter sequence was revealed to be conserved (Liu et al. 2014; Taylor et al. 2019). The pattern of cross-species synteny facilitated also the assembly of the *L. angustifolius* pseudochromosomes, providing anchors for scaffold order and orientation in the regions where the marker resolution was insufficient due to low recombination rate (Hane et al. 2017). This assembly has been further improved with the aid of an ultra-high-density genetic map containing 34574 sequence-defined markers (Zhou et al. 2018).

Molecular studies on white lupin based on comparative mapping approaches have been hampered for many years because the published linkage maps carried a relatively small number of sequenced markers (Croxford et al. 2008; Phan et al. 2007; Vipin et al. 2013). The most recent linkage map of white lupin with 3669 sequenced markers highlighted the collinearity between *L. angustifolius* and *L. albus* genomes and provided novel possibilities for map-based gene cloning (Książkiewicz et al. 2017). This tool was further exploited to identify candidate genes involved in white lupin early flowering (Rychel et al. 2019). The information on highly conserved synteny between *L. albus* and *L. angustifolius* genomes was also harnessed in the present study to design new markers tagging low alkaloid *pauper* locus.

### Markers and genes for low alkaloid content in lupins

Alkaloid content as a major potentially toxic anti-nutritional factor was thoroughly investigated during the lupin domestication process, and numerous low alkaloid accessions were selected in all three crop Old World lupin species: *L. angustifolius*, *L. albus*, and *L. luteus* (Hackbarth 1957; Hackbarth 1961; Hackbarth and Troll 1956; Święcicki 1986; Święcicki and Jach 1980; Święcicki and Święcicki 1995). Significant progress in the determination of low alkaloid lines has been also achieved in the main New World lupin crop, *L. mutabilis* (Galek et al. 2017). Among lupins, *L. angustifolius* has been subjected to the most advanced studies on genetic and molecular factors affecting quinolizidine alkaloid biosynthesis. Three major recessive low-alkaloid alleles were identified in *L. angustifolius* germplasm, namely *iucundus*, *depressus*, and *esculentus*; however, only *iucundus* was widely introduced into breeding programs (Święcicki and Święcicki 1995). *L. angustifolius* RIL mapping population developed from the cross of 83A:476 (maternal, sweet, domesticated) and P27255 (paternal, bitter, wild) enabled genetic localization of *iucundus* locus. However, this trait revealed high distortion from the expected 1:1 segregation ratio, evidenced by the chi-square *P* value of 0.008 (Boersma et al. 2005). *Iucundus* was localized in all versions of the *L. angustifolius* linkage map but was surrounded only by MFLP-derived markers lacking sequence information (Boersma et al. 2005; Kamphuis et al. 2015; Nelson et al. 2010). To provide a DNA marker tightly linked to *iucundus* (~ 0.9 cM), a separate study was performed involving 20 lines and 320 MFLP fingerprints (Li et al. 2011). Novel markers closely related to the *iucundus* were developed during genome sequencing attempts; however, no candidate gene was hypothesized (Hane et al. 2017; Zhou et al. 2018). Recently, transcriptome-based studies revealed high correlation of alkaloid content with leaf tissue expression levels of genes encoding lysine/ornithine decarboxylase (*LaL/ODC*), copper amine oxidase (*LaCAO*), acyltransferase (*LaAT*), berberine bridge enzyme (*LaBBE-like*), and major latex-like proteins (*LaMLP1-like*, *LaMLP2-like*, and *LAMLP4-like*) (Frick et al. 2018; Yang et al. 2017). The group of *L. angustifolius* quinolizidine alkaloid biosynthesis genes is putatively regulated by an APETALA2/ethylene responsive transcription factor, which was evidenced by linkage mapping and transcriptome profiling as a strong candidate for *iucundus* (Kroc et al. 2019).

In *L. luteus*, four low-alkaloid alleles were identified, including *dulcis*, *amoenus*, *liber* (von Sengbusch 1942), and *v* (Gustafsson and Gadd 1965). Forms with alkaloid content below 0.05% were developed (Święcicki and Jach 1980). Molecular resources for this species are very limited and include two transcriptome assemblies derived from independent studies and the set of insertion/deletion markers developed by next-generation sequencing of genomic reduction libraries (Glazinska et al. 2017; Osorio et al. 2018; Parra-González et al. 2012). The lack of mapping populations and linkage maps considerably impeded research on genes underlying low alkaloid content in this species.

Genetic studies involving *L. albus* germplasm resulted in the identification of several loci underlying low alkaloid content: *pauper*/*primus*/*tercius*, *exiguus*, *nutricius*, *mitis*, *suavis*, *reductus*, and *minutus* (Hackbarth 1957; Hackbarth 1961; Harrison and Williams 1982; Porsche 1964; Šatović 1993; Troll 1958). *Pauper* was widely exploited for breeding; *exiguus* and *nutricius* were used occasionally, whereas other loci remained untapped (Harrison and Williams 1982; Šatović 1993). First two linkage maps addressing *pauper* segregation did not provide any marker closely related to this gene (Phan et al. 2007; Vipin et al. 2013). With the aid of the MFLP technique, a PCR-based PauperM1 marker tagging *pauper* locus by 1.4 cM was developed (Lin et al. 2009). However, due to the small length difference between the alleles and the production of some background stutter bands, analysis of this marker required tedious and time-consuming sequencing gel electrophoresis.

### A step towards identification of the gene underlying *pauper* locus

In this paper, the PauperM1 marker was improved to a CAPS marker addressing two closely located SNPs, recognized by two different enzymes. Enzyme *HhaI* is expected to cut the bitter allele, whereas *Hin*fI—the sweet one. Such an approach minimizes the risk of false-positive and false-negative scores resulting from non-occurrence of cleavage due to reaction preparation issues. In the present study, six SNP markers generated by genotyping-by-sequencing (Książkiewicz et al. 2017) were transformed to PCR-based markers using CAPS and dCAPS approaches (Konieczny and Ausubel 1993; Neff et al. 1998). It is widely adapted strategy for scoring SNP markers obtained by high-throughput sequencing (Shavrukov 2016). Several tools were developed, allowing design of CAPS and/or dCAPS markers on one-by-one basis (dCAPS Finder, BlastDigester, SNP2CAPS, SGN CAPS Designer) or as a high-throughput automated process (CAPS/dCAPS Designer) (Ilic et al. 2004; Li et al. 2018; Neff et al. 2002; Thiel et al. 2004). For routine implementation, other PCR allelic discrimination technologies are considered, including rhAmp, TaqMan, or KASP assays (Broccanello et al. 2018). Indeed, a Fluidigm nanofluidic array genotyping platform has been exploited to formulate *L. angustifolius* SNP array and provide markers for linkage mapping and genome assembly (Hane et al. 2017; Kamphuis et al. 2015; Yang et al. 2013; Zhou et al. 2018).

One of the newly developed markers, LAGI01_35805_F1_R1, was revealed to have higher applicability for *pauper* marker-assisted selection than the previously published PauperM1 marker (Lin et al. 2009), evidenced by higher values of all coefficients calculated (simple matching, Rogers-Tonimoto, Pearson product-moment correlation). However, false-positive scores were revealed for four lines (constituting 2.5% of analyzed plant materials). All these lines were primitive accessions or landraces, including a line with the highest dry weight seed alkaloid content in the collection, 95015 “San Felices” (Kroc et al. 2017). Such an observation may indicate that the *pauper* locus gene is different than the gene represented by the LAGI01_35805_F1_R1 marker sequence. It is also possible that the LAGI01_35805 is derived from the true *pauper* locus gene but the SNP recognized by this marker is not the functional mutation causing low alkaloid content. LAGI01_35805 is a homolog of *L. angustifolius* Lup021586 gene, annotated as the *LaAT* gene (AB581532.1) (Książkiewicz et al. 2017). *LaAT* is a representative of BAHD acyl-CoA-dependent acyltransferase superfamily and was shown to be highly expressed in the leaves of quinolizidine alkaloid-producing *L. angustifolius* plants but undetectable in the sweet ones (Bunsupa et al. 2011). However, in *L. angustifolius*, the function of low alkaloid *iucundus* gene is assigned rather to a regulatory agent (a transcription factor) than to an enzyme directly involved in alkaloid biosynthesis (Kroc et al. 2019). Nevertheless, *L. angustifolius* and *L. albus* have a relatively different pattern of alkaloid compound variation and partially differ by major component influencing total alkaloid content (Boschin et al. 2008; Kamel et al. 2016; Kroc et al. 2017). It was suggested that functions of *iucundus* and *pauper* genes may be distinct because these species have different lysine profiles among wild and sweet accessions (Frick et al. 2017). As expected, negative LAGI01_35805_F1_R1 scores were obtained for four low alkaloid lines carrying *exiguus* gene (95422 “Start”, 95480 “Nelly”, 95513 “Butan” derived from Start × Wat, 95454 “Tombowskij Skorospielyj”) and one carrying *nutricius* gene (95509 “Nahrquell”) (Hackbarth 1957; Hackbarth 1961; Harrison and Williams 1982; Porsche 1964; Šatović 1993; Troll 1958; Stawiński S. unpublished). Additionally, negative LAGI01_35805_F1_R1 scores were revealed for six sweet lines with unknown low-alkaloid donor (95175 “R-84141”, 95433 “Dniepr”, 95160 “R-243”, 95166 “SF-479”, 95445 “Lutrop,” and 95449 “Buttercup”). These lines may carry *exiguus*, *nutricius*, or other low-alkaloid genes.

In the sister crop species, narrow-leafed lupin, the breeding process has been considerably facilitated by markers which were developed to select key agronomic traits and subsequently implemented in Australian breeding programs. These include early flowering (KuHM1, LanFTc1_INDEL) (Boersma et al. 2007a; Nelson et al. 2017), reduced pod shattering (TaLi, TaM1, TaM2, LeM1, LeM2, LeLi) (Boersma et al. 2007b; Boersma et al. 2009; Li et al. 2010; Li et al. 2012c), low alkaloid profile (iucLi) (Li et al. 2011), soft seediness (marker MoLi) (Li et al. 2012b), and resistance to diseases caused by pathogenic fungi, including anthracnose (AntjM1, AntjM2, AnManM1) (Yang et al. 2004; Yang et al. 2008a; You et al. 2005) and Phomopsis stem blight (PhtjM1, PhtjM2, Ph258M1, Ph258M2) (Yang et al. 2002). Numerous studies revealed that the further improvement of white lupin as a crop will require incorporation of rare alleles, such as resistance to anthracnose found only in Ethiopian lines, from wild landraces which are bitter and late flowering (Adhikari et al. 2009; Adhikari et al. 2013; Phan et al. 2007). Reselection of agronomic traits in the progeny could be greatly facilitated by the use of markers targeting particular domestication genes. Such a model was established for the narrow-leafed lupin (Cowling et al. 2009). Markers for low alkaloid *pauper* locus developed in this study, together with those recently published for early flowering (Rychel et al. 2019), address this requirement and constitute a versatile array for white lupin molecular breeding.

## Electronic supplementary material

Supplementary File 1 Lines used for validation of white lupin markers from *pauper* region. (XLSX 20 kb)

Supplementary File 2 Segregation data revealed in mapping population for white lupin markers from *pauper* region. (20 kb)

Supplementary File 3 Visualization of polymorphism for white lupin markers from *pauper* region. (DOCX 1753 kb)

Supplementary File 4 Genotype scores revealed in white lupin collection lines for white lupin markers from *pauper* region. (XLSX 23 kb)
