# Supplementary material for: Development of gene-based molecular markers tagging low alkaloid pauper locus in white lupin (Lupinus albus L.)
Source: J Appl Genet. 2019 Aug 13;60(3):269–81. doi: 10.1007/s13353-019-00508-9 (PMC6803572; doi:10.1007/s13353-019-00508-9)
Supplement: Supplementary file 3 — Visualization of polymorphism for white lupin markers from pauper region. (DOCX 1753 kb) [file 13353_2019_508_MOESM3_ESM.docx]

Development of gene-based molecular markers tagging low alkaloid *pauper* locus in white lupin (*Lupinus albus* L.)

Sandra Rychel^1^, Michał Książkiewicz^1^

^1^ Institute of Plant Genetics, Polish Academy of Sciences, Strzeszyńska 34, 60-479 Poznań

**Supplementary File 3. Visualization of polymorphism for white lupin markers from pauper region.**

PauperM1_F_R_*Hinf*I

**GeneRuler DNA
Ladder Mix**

3000 bp

1000 bp

500 bp

200 bp


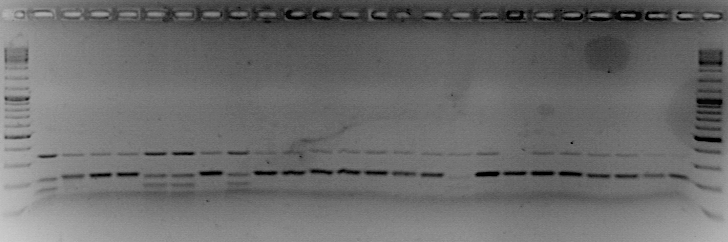


**1 3 3 3 1 1 3 1 3 3 3 3 3 3 3 1 3 3 3 3 3 3 3 3**

**M 221 222 223 224 225 226 227 228 229 230 231 232 233 234 237 238 239 240 241 243 244 245 246 247 M**

PauperM1_F_R_*Hha*I


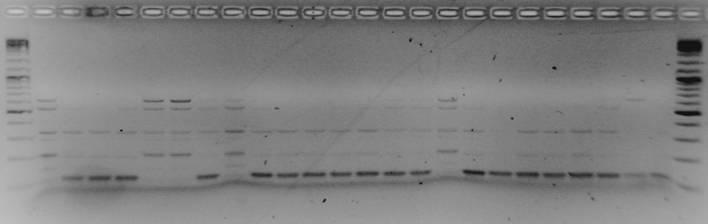


**M 221 222 223 224 225 226 227 228 229 230 231 232 233 234 237 238 239 240 241 243 244 245 246 247 M**

**1 3 3 3 1 1 3 1 3 3 3 3 3 3 3 1 3 3 3 3 3 3 3 3**

**GeneRuler DNA
Ladder Mix**

3000 bp

1000 bp

500 bp

200 bp

TP16854_FD_R

**GeneRuler DNA
Ladder Mix**

3000 bp

1000 bp

500 bp

200 bp


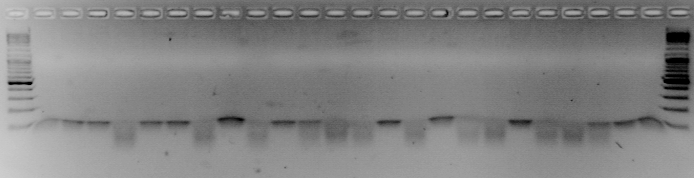


**M 221 222 223 224 225 226 227 228 229 230 231 232 233 234 237 238 239 240 241 243 244 245 246 247 M**

**1 1 1 3 1 1 3 1 3 1 1 3 3 1 3 1 3 3 1 3 3 3 1 1**

LAGI01_35805_F2_R2


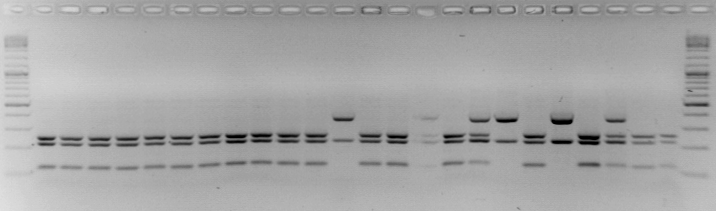


**1 1 1 1 1 1 1 1 1 1 1 3 3 1 2 1 2 3 1 3 1 2 1 1**

**M 221 222 223 224 225 226 227 228 229 230 231 232 233 234 237 238 239 240 241 243 244 245 246 247 M**

**GeneRuler DNA
Ladder Mix**

3000 bp

1000 bp

500 bp

200 bp

LAGI01_35805_F1 _R1

**GeneRuler DNA
Ladder Mix**

3000 bp

1000 bp

500 bp

200 bp


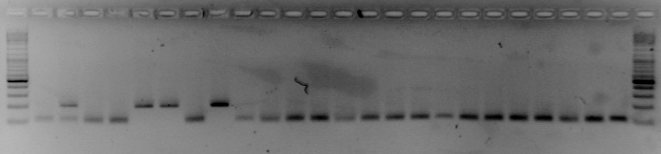


**3 2 3 3 1 1 3 1 3 3 3 3 3 3 3 3 3 3 3 3 3 3 3 3**

**M 221 222 223 224 225 226 227 228 229 230 231 232 233 234 237 238 239 240 241 243 244 245 246 247 M**

LAGI01_49436_F2_R2

**GeneRuler DNA
Ladder Mix**

3000 bp

1000 bp

500 bp

200 bp


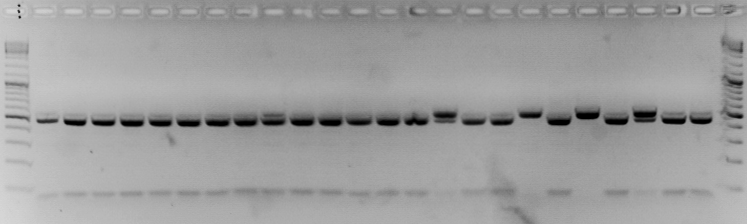


**1 1 1 1 1 1 1 1 1 1 1 1 1 1 2 1 1 3 1 3 1 2 1 1**

**M 221 222 223 224 225 226 227 228 229 230 231 232 233 234 237 238 239 240 241 243 244 245 246 247 M**

LAGI54458_F2_R1

**GeneRuler DNA
Ladder Mix**

3000 bp

1000 bp

500 bp

200 bp


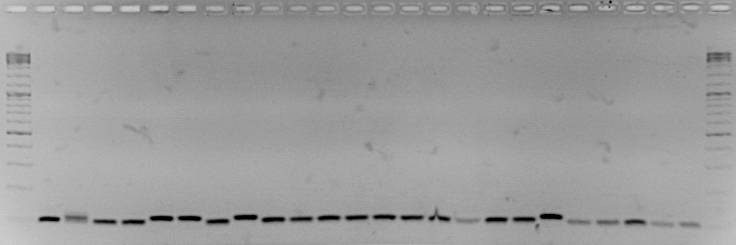


**3 2 3 3 1 1 3 1 3 3 3 3 3 3 3 3 3 3 1 3 3 3 3 3**

**M 221 222 223 224 225 226 227 228 229 230 231 232 233 234 237 238 239 240 241 243 244 245 246 247 M**

TP447859_F_R

**GeneRuler DNA
Ladder Mix**

3000 bp

1000 bp

500 bp

200 bp


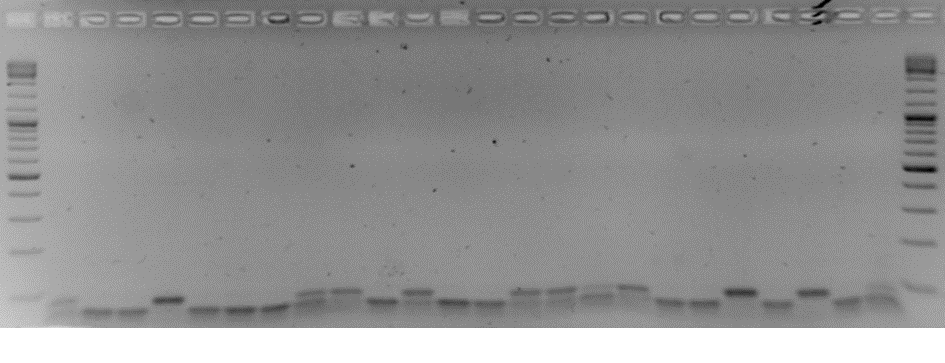


**2 1 1 3 1 1 1 2 3 1 2 1 1 3 3 1 3 1 1 3 1 3 1 1**

**M 221 222 223 224 225 226 227 228 229 230 231 232 233 234 237 238 239 240 241 243 244 245 246 247 M**

TP22150_F_R

**GeneRuler DNA
Ladder Mix**

3000 bp

1000 bp

500 bp

200 bp


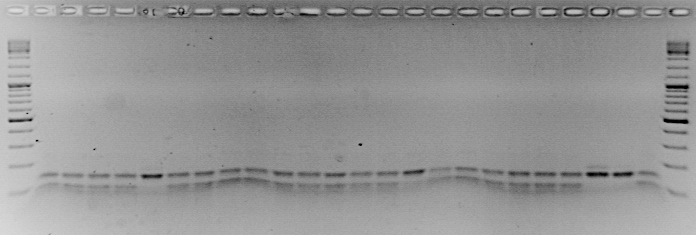


**1 1 1 1 3 1 1 1 1 1 1 1 1 1 3 1 1 1 1 1 1 3 3 1**

**M 221 222 223 224 225 226 227 228 229 230 231 232 233 234 237 238 239 240 241 243 244 245 246 247 M**

ESD4-F7

**GeneRuler DNA
Ladder Mix**

3000 bp

1000 bp

500 bp

200 bp


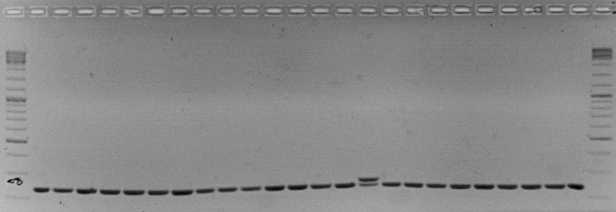


**1 1 1 1 1 1 1 1 1 1 1 1 1 1 2 1 1 1 1 1 1 1 1 1**

**M 221 222 223 224 225 226 227 228 229 230 231 232 233 234 237 238 239 240 241 243 244 245 246 247 M**

ESD4-F8

**GeneRuler DNA
Ladder Mix**

3000 bp

1000 bp

500 bp

200 bp


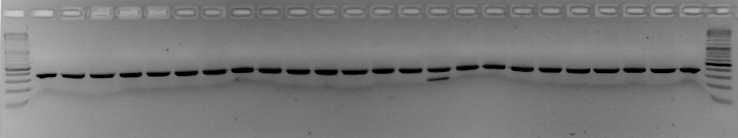


**1 1 1 1 1 1 1 1 1 1 1 1 1 1 2 1 1 1 1 1 1 1 1 1**

**M 221 222 223 224 225 226 227 228 229 230 231 232 233 234 237 238 239 240 241 243 244 245 246 247 M**

TP309728_F_R

**GeneRuler DNA
Ladder Mix**

3000 bp

1000 bp

500 bp

200 bp


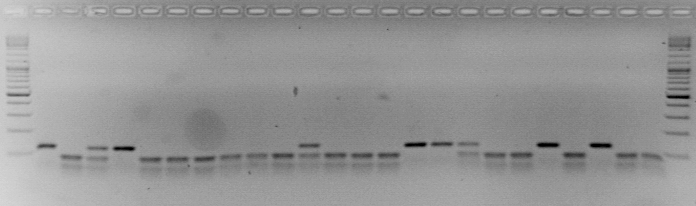


**M 221 222 223 224 225 226 227 228 229 230 231 232 233 234 237 238 239 240 241 243 244 245 246 247 M**

**3 1 2 3 1 1 1 1 1 1 2 1 1 1 3 3 2 1 1 3 1 3 1 1**

TP70046_F_RD

**GeneRuler DNA
Ladder Mix**

3000 bp

1000 bp

500 bp

200 bp


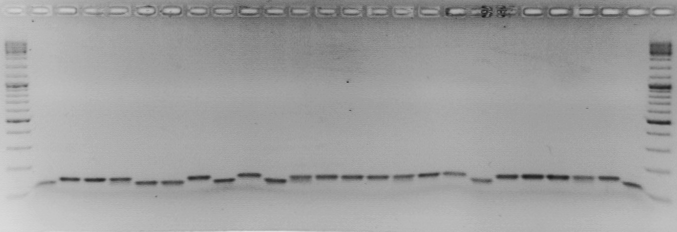


**M 221 222 223 224 225 226 227 228 229 230 231 232 233 234 237 238 239 240 241 243 244 245 246 247 M**

**1 3 3 3 1 1 3 1 3 1 3 3 3 3 3 3 3 1 3 3 3 3 3 1**

TP30216_F_R

**GeneRuler DNA
Ladder Mix**

3000 bp

1000 bp

500 bp

200 bp


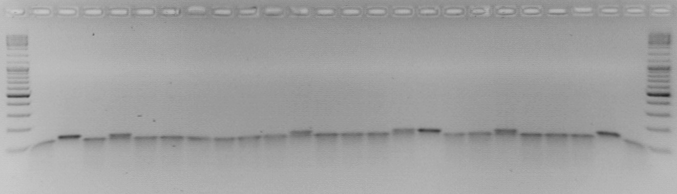


**M 221 222 223 224 225 226 227 228 229 230 231 232 233 234 237 238 239 240 241 243 244 245 246 247 M**

**1 3 1 2 1 1 1 1 1 1 2 1 1 1 2 3 1 1 2 1 1 1 3 1**

M - Thermo Scientific GeneRuler DNA Ladder Mix #SM0332

1 - Kiev Mutant allele

2 - heterozygote

3 - P27174 allele

White lupin lines

| ID | Line name | Accession |
| --- | --- | --- |
| 221 | Tremoco Beja | 95264 |
| 222 | Pop.Setubal | 95266 |
| 223 | BG-9787 | 95269 |
| 224 | BGR6305 | 95275 |
| 225 | Bialorus-1 | 95414 |
| 226 | Bialorus-2 | 95415 |
| 227 | Bialorus-3 | 95416 |
| 228 | Volodia | 95431 |
| 229 | Primorskij | 95432 |
| 230 | Dniepr | 95433 |
| 231 | Silosnyj | 95439 |
| 232 | Lublanc | 95443 |
| 233 | Horizont | 95456 |
| 234 | ILCA13665 | 95518 |
| 237 | ILCA13677 | 95521 |
| 238 | Population-776 | 95606 |
| 239 | Population-804 | 95608 |
| 240 | Mansilla de los Mulas | 95007 |
| 241 | Magarraz-2 | 95012 |
| 243 | Population-8057 | 95062 |
| 244 | Azorski | 95095 |
| 245 | Population-84075 | 95128 |
| 246 | Population-187 | 95138 |
| 247 | R-243 | 95160 |
